# Neuropeptides as the Shared Genetic Crosstalks Linking Periodontitis and Major Depression Disorder

**DOI:** 10.1155/2021/3683189

**Published:** 2021-10-21

**Authors:** Changsheng Sun, Jiatong Han, Yixin Bai, Zhaowei Zhong, Yingtao Song, Yu Sun

**Affiliations:** ^1^Department of Oral and Maxillofacial Surgery, Dental Hospital, The First Affiliated Hospital of Harbin Medical University, 23 Youzheng Road, Nangang District, Harbin, 150001 Heilongjiang, China; ^2^Department of Prosthodontics, Dental Hospital, The First Affiliated Hospital of Harbin Medical University, 23 Youzheng Road, Nangang District, Harbin, 150001 Heilongjiang, China

## Abstract

**Background:**

The aim of this study was at investigating the association between major depressive disorder (MDD) and periodontitis based on crosstalk genes and neuropeptides.

**Methods:**

Datasets for periodontitis (GSE10334, GSE16134, and GSE23586) and MDD (GSE38206 and GSE39653) were downloaded from GEO. Following batch correction, a differential expression analysis was applied (MDD: ∣log2FC | >0 and periodontitis ∣log2FC | ≥0.5, *p* < 0.05). The neuropeptide data were downloaded from NeuroPep and NeuroPedia. Intersected genes were potential crosstalk genes. The correlation between neuropeptides and crosstalk genes in MDD and periodontitis was analyzed with Pearson correlation coefficient. Subsequently, regression analysis was performed to calculate the differentially regulated link. Cytoscape was used to map the pathways of crosstalk genes and neuropeptides and to construct the protein-protein interaction network. Lasso regression was applied to screen neuropeptides, whereby boxplots were created, and receiver operating curve (ROC) analysis was conducted.

**Results:**

The MDD dataset contained 30 case and 33 control samples, and the periodontitis dataset contained 430 case and 139 control samples. 35 crosstalk genes were obtained. A total of 102 neuropeptides were extracted from the database, which were not differentially expressed in MDD and periodontitis and had no intersection with crosstalk genes. Through lasso regression, 9 neuropeptides in MDD and 43 neuropeptides in periodontitis were obtained. Four intersected neuropeptide genes were obtained, i.e., ADM, IGF2, PDYN, and RETN. The results of ROC analysis showed that IGF2 was highly predictive in MDD and periodontitis. ADM was better than the other three genes in predicting MDD disease. A total of 13 crosstalk genes were differentially coexpressed with four neuropeptides, whereby FOSB was highly expressed in MDD and periodontitis.

**Conclusion:**

The neuropeptide genes ADM, IGF2, PDYN, and RETN were intersected between periodontitis and MDD, and FOSB was a crosstalk gene related to these neuropeptides on the transcriptomic level. These results are a basis for future research in the field, needing further validation.

## 1. Background

Major depressive disorder (MDD) is a prevalent disease worldwide, showing a prevalence around 5% and an incidence of approximately 3%, with differences between countries [1]. Thereby, MDD is the most prevalent mental disorder in the world [2]. MDD is a disabling condition, worsening quality of life, limiting patients in their everyday activities, and resulting in a remarkable morbidity of patients [2]. Additionally, MDD is often related with comorbidities, worsening the general outcome of respective patients [2]. It has been indicated that MDD is potentially related to changes in numerous biological pathways and systems, including the gastrointestinal tract, immune system, hormones, and oxidative stress [3, 4]. In this context, an increased understanding of the etiopathogenesis of MDD and related cofactors is of high clinical interest to reveal potential therapeutic strategies in the future [3]; however, it is difficult to reveal any causalities between other diseases, biomarkers, and MDD in the clinical context [5].

One potentially associated disease is periodontitis, which is an inflammatory, multifactorial infectious disease of the tooth-surrounding tissues [6, 7]. Generally, severe periodontitis affects about 11% of the world population, making it a highly prevalent chronic disease [8]. Different systematic reviews and meta-analyses are available, which indicated an association between periodontitis and MDD, although results are quite heterogeneous regarding the magnitude of correlation [6, 9, 10]. A Brazilian birth cohort study revealed a risk ratio of patients with MDD to suffer from periodontitis of 1.19 [11]. Another observational study including 60000 individuals over a 10-year follow-up showed a higher incidence of MDD in periodontally diseased individuals, supported by a hazard ratio of 1.73 [12]. Thereby, it is of interest, whether there is a primary association between periodontitis and MDD based on psychosocial mechanisms or even a causal link [13]. The potential role of neuroinflammation induced by chronic inflammatory periodontal diseases, especially due to the expression of proinflammatory cytokines, has been discussed as potential causal link [13, 14]. Thereby, a role of periodontal pathogenic bacteria has been presumed, which is supported by the induction of neuroinflammation by *Fusobacterium nucleatum* [15].

Although these approaches are interesting and appear plausible, there is more research needed to support the causal interlink between periodontitis and MDD [13]. One potential approach could be the application of bioinformatics to reveal a potential interlink on the transcriptomic level; this has been established for other potential interlinks, e.g., between periodontitis and Alzheimer's disease [16]. Thereby, the potential integration of crosstalk genes, alongside with neuropeptides could be a promising approach to gain insight into the interrelationship between MDD and periodontitis. Thereby, neuropeptides could be of particular interest, because they are involved in a magnitude of processes and were not examined in the context of MDD and periodontitis, yet. Accordingly, this current study is aimed at investigating the association between MDD and periodontitis based on crosstalk genes and their potential link to neuropeptides in these two diseases.

## 2. Materials and Methods

### 2.1. Data Download

The expression data of periodontitis and MDD was downloaded from GEO (https://www.ncbi.nlm.nih.gov/geo/). For periodontal disease (PD), gingival tissue samples were used, whereby datasets GSE10334, GSE16134, and GSE23586 were obtained. Whole-blood peripheral blood mononuclear cell (PBMC) samples were used for MDD, where two datasets, i.e., GSE38206 and GSE39653, are obtained. For the dataset GSE38206, the experimental group id MDE-P-0W and the control group id C-0W were included. Data from the experimental group and control group at 8 weeks of follow-up were not used. For GSE39653, MDD and healthy control (HC) samples were included. The datasets of MDD and PD are shown in Table 1.

### 2.2. Data Preprocessing and Differential Expression Analysis

Firstly, a probe ID was converted into Gene Symbol according to their platform information. For the data of multiple probes corresponding to the same gene, the mean value of the sample was used as its expression value.

Then, all samples from each disease were combined for PD and MDD expression profiles. To reduce the differences of included samples, a batch correction using the ComBat method in the “sva” package of R project was applied.

The “limma” of R language was used for differential expression analysis of the corrected MDD and PD datasets. For MDD, the genes with a *p* value < 0.05 and ∣log2FC | >0 were differentially expressed genes (DEGs). Thereby, upregulation was evaluated if log2FC > 0, while log2FC < 0 was the cutoff for downregulated genes. The genes with a *p* value < 0.05 and ∣log2FC | ≥0.5 in PD were considered as differentially expressed genes. The log2FC ≥ 0.5 were the upregulated genes and log2FC ≤ −0.5 were the downregulated genes.

### 2.3. Neuropeptide Download

The neuropeptide data were downloaded from the database NeuroPep (http://www.neuropeptides.nl/) and NeuroPedia (http://proteomics.ucsd.edu/Software/NeuroPedia.html). After merging the two databases, a total of 102 neuropeptide genes were obtained.

### 2.4. Crosstalk Gene Screening

The intersection of differentially expressed genes obtained from MDD and PD was taken, while the intersection genes were the potential crosstalk genes. To analyze the function of these crosstalk genes, clusterProfiler in R language was used for GO Biological Process and KEGG Pathway enrichment analysis and functions with *p* value < 0.05 were significant.

### 2.5. Differential Coexpression Regulation Links of Crosstalk Genes and Neuropeptides

First, it was analyzed whether there were crosstalk genes within the neuropeptides. This step revealed that none of the genes was both crosstalk gene and neuropeptide. Then, the correlation between neuropeptides and crosstalk genes in MDD and PD was analyzed. Therefore, Pearson correlation coefficient was calculated to assess the direct correlation between neuropeptides and crosstalk genes. The crosstalk gene-neuropeptide pair with the correlation coefficient (CC) absolute value greater than 0.5 (∣CC | >0.5) was considered as significant relationship pairs. In order to further analyze the regulatory effect of significant relationships between crosstalk genes and neuropeptides, the expression values of significant relationships between genes in diseased and healthy groups of MDD and PD were examined, respectively. Subsequently, regression analysis was performed to calculate the differentially regulated link (DRL).

In the data of regression analysis, the regulatory effect of crosstalk genes with neuropeptides was assessed, whereby the expression of crosstalk genes acted as *x* and expression of neuropeptides as *y* in the formula *y* ~ *βx* + *β*_0_.

By calculating this formula, it is possible to obtain the regression coefficients of the crosstalk gene-neuropeptide relationship (cross_*i*_ − neurop_*j*_) in the diseased group and healthy control group of MDD and PD, respectively. Then, the sum of the standard deviations of the relationships in the case group and healthy control group was calculated for both MDD and PD, respectively. The correlation coefficients in all samples of MDD and PD were calculated. Finally, the regulation coefficients of the relationship in differential coexpression genes were calculated according to the formula shown below.

(1) $$\begin{array}{c} {\text{DRL}}_{i,j}=\frac{\beta_{\text{case}}-\beta_{\text{control}}}{\sqrt{{sd}_{\text{case}}^{2}+{sd}_{\text{control}}^{2}+CC{\left(i,j\right)}_{\text{all}}}}. \end{array}$$

A positive DRL indicates a consistent regulation of the crosstalk-neuropeptide relationship in case and control samples, while both are either inhibiting or promoting. If the value is negative, the relationship is opposite in case and control regulation modes.

### 2.6. Functional Analysis of Crosstalk Genes and Neuropeptides

The human pathway-gene pairs were downloaded from KEGG (https://www.kegg.jp/), and the pathways corresponding to crosstalk genes and neuropeptides were extracted. It has been further evaluated whether the pathway contains both crosstalk genes and neuropeptides, followed by an analysis of the pathways regulated by crosstalk genes and neuropeptides. These coacting pathways may be the key pathways for the interaction of the two genes. Cytoscape was used to map the pathways of crosstalk genes and neuropeptides.

### 2.7. Analysis of Crosstalk Genes and Neuropeptides in the Protein Interaction Network

For experimental verification of protein-protein interactions (PPI), data were downloaded for MINT (http://mint.bio.uniroma2.it/mint/Welcome.do), HPRD (http://www.hprd.org/index_html), BIOGRID (http://thebiogrid.org/), DIP (http://dip.doe-mbi.ucla.edu/dip/Main.cgi), menthe (http://mentha.uniroma2.it/index.php), PINA (http://cbg.garvan.unsw.edu.au/pina/), InnateDB (http://www.innatedb.com/), and Instruct (http://instruct.yulab.org/index.html). PPI pairs of crosstalk genes and neuropeptides were then extracted. In order to further analyze the relationship between crosstalk genes and neuropeptides in the system biological network, one-step PPI was extended according to the relationship pairs. An indirect pair (cross-other-neuropeptide) that regulated both crosstalk genes and neuropeptides was extracted. Then Cytoscape software was used to construct the PPI network. The relationship links were displayed in the network and the topology properties were analyzed.

### 2.8. Neuropeptide Screening

The expression values of all neuropeptides in MDD and PD were extracted and the Lasso regression analysis was applied to screen neuropeptides. The expression values of specific neuropeptides in PD and MDD were extracted, boxplots were created, and receiver operating curve (ROC) analysis was conducted. In addition, the differential regulatory weights of these specific neuropeptides and their highly correlated crosstalk genes were extracted in MDD and PD. Finally, the interaction pathways for these specific neuropeptides and the corresponding highly correlated crosstalk genes were evaluated, the neuropeptides were associated with the crosstalk genes and the pathways, and the functions influenced by neuropeptides and crosstalk genes were analyzed.

## 3. Results

### 3.1. Data Preprocessing

**Figure 1:**
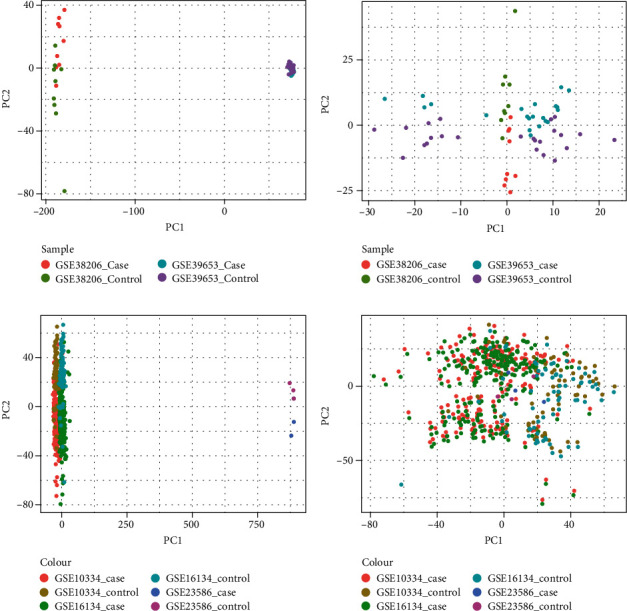
(a, b) PCA analysis results of MDD batches before and after rectification; (c, d) PCA analysis results of PD batch before and after correction.

After data combination and batch correction, a dataset of MDD and PD was created, wherein MDD contains 30 case and 33 control samples, while PD contains 430 case and 139 control samples. At the same time, PCA analysis results of data before and after correction (Figure 1) for the two diseases were reviewed.

### 3.2. Differential Expression Analysis

**Figure 2:**
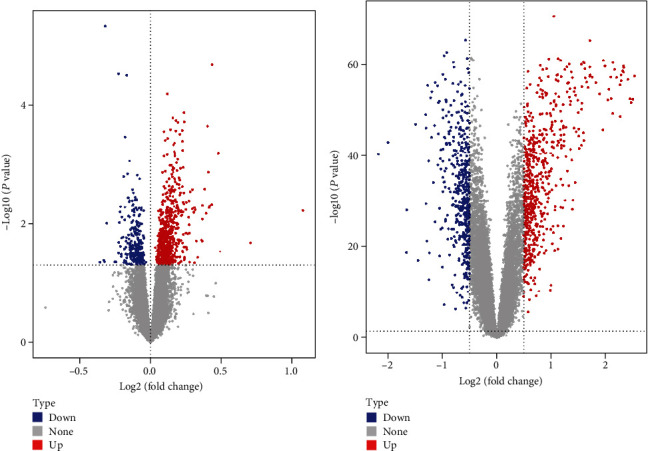
Volcano maps of differentially expressed genes in (a) MDD and (b) PD.

According to the analysis results, the differentially expressed genes were screened and the volcano diagram was used to show the cutoff screening of differentially expressed genes (Figures 2(a) and 2(b)). The number of differential expressed genes obtained is shown in Table 2.

### 3.3. Crosstalk Gene Screening

**Figure 3:**
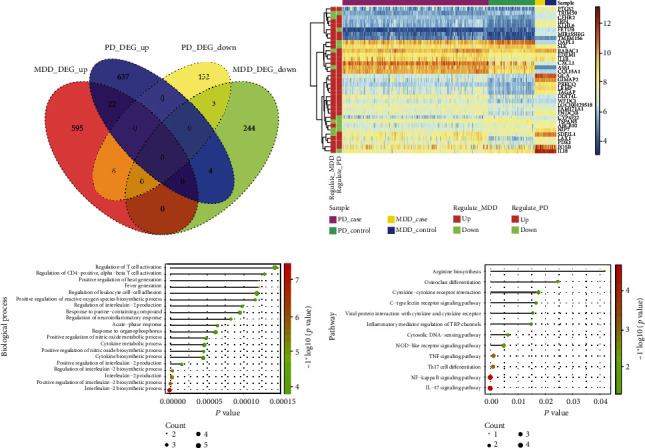
Regulation function of crosstalk genes. (a) Venn maps of differentially expressed genes obtained from MDD and PD. (b) Heat maps of 35 crosstalk genes in MDD and PD. (c) Top 20 biological processes of significant enrichment of crosstalk genes. (d) All significant KEGG pathways were regulated by significant crosstalk genes.

The intersection of differentially expressed genes obtained from MDD and PD were the respective crosstalk genes (Figure 3(a)). A total of 35 crosstalk genes were obtained (Figure 3(b)). clusterProfiler package of R language was used for functional enrichment analysis of these 35 crosstalk genes (significance level *p* value < 0.05).  Figure 3(c) shows the biological processes in which the 35 crosstalk genes were mainly involved (Figure 3(c)). The 35 crosstalk genes mainly regulated the IL-17 signaling pathway, NF-Kappa B signaling pathway, and TNF signaling pathway (Figure 3(d)).

### 3.4. The Links between Crosstalk Genes and Neuropeptides

**Figure 4:**
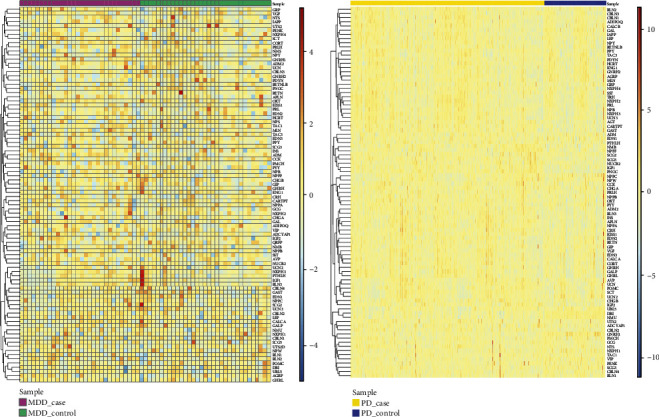
Heat maps of neuropeptide expression in (a) MDD and (b) PD.

A total of 102 neuropeptides were extracted from the database, and the expression values of these genes in MDD and PD were further extracted to construct the heat map (Figure 4). The results showed that neuropeptides were not differentially expressed in MDD and PD. Moreover, crosstalk genes and neuropeptides had no intersection, indicating that no gene was revealed to be both crosstalk gene and neuropeptide.

**Figure 5:**
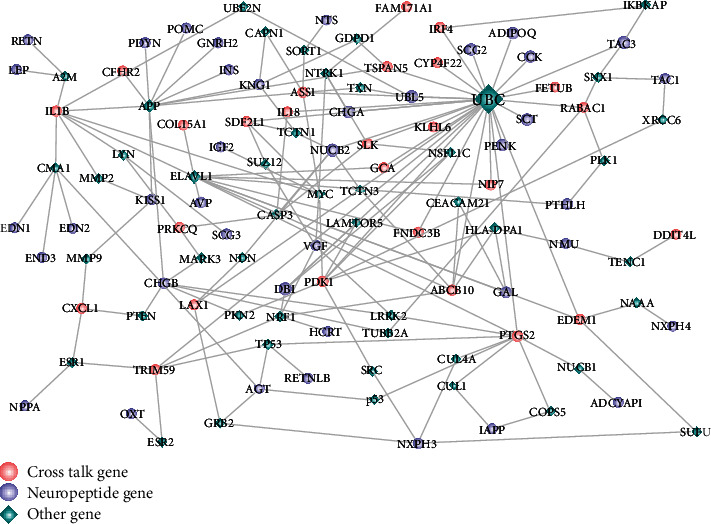
Crosstalk genes and neuropeptides in the PPI-network. In the network, crosstalk genes and neuropeptides are linked by bridging genes.

In order to further analyze the role of crosstalk genes and neuropeptides in the entire biological network, the relationship pairs of the direct interaction between crosstalk genes and neuropeptides were extracted according to the known PPI relationship pairs. As a result, no direct interaction pairs were obtained. According to the interaction proteins of crosstalk genes, a step extension and subsequent screening of the extended proteins as genes of neuropeptides obtained a total of 164 relationship pairs. Then Cytoscape software was used to construct the PPI network (Figure 5).

Topological properties of the top 20 genes were screened out according to the degree in descending order. The results are shown in Table 3.

From the PPI network, neuropeptide genes NUCB2, DBI, and UBL5 could interact with more genes, thus indirectly interacting with crosstalk genes.

### 3.5. Differential Coexpression Regulation Links between Crosstalk Genes and Neuropeptides

**Figure 6:**
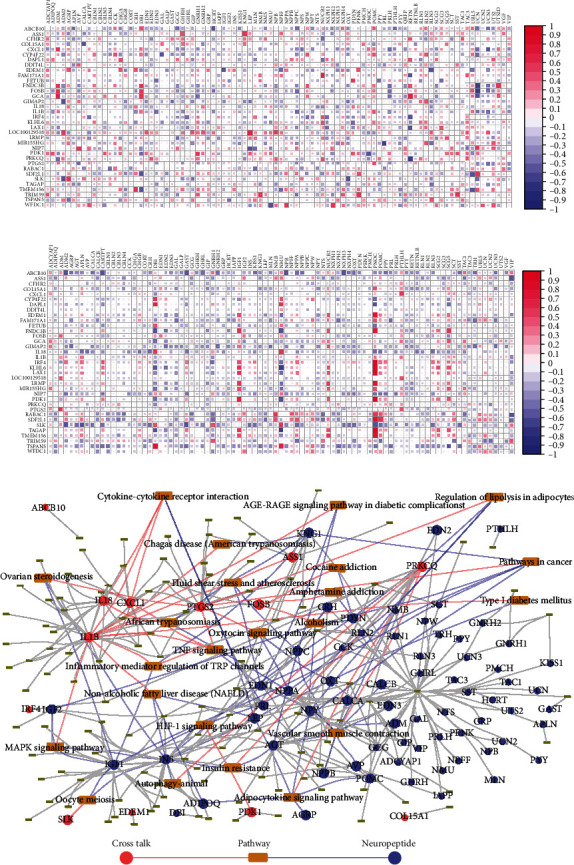
Correlation between crosstalk genes and neuropeptides in disease groups of (a) MDD and (b) PD and (c) crosstalk gene-pathway-neuropeptide network.

To identify the function of neuropeptides in MDD and PD, the correlation between crosstalk genes and neuropeptides in MDD and PD was assessed firstly.  Figure 6 shows the correlation between crosstalk genes and neuropeptides in the case group of MDD and PD (Figure 6).

The significant correlation pairs in the case and control groups of MDD and PD were selected, and the regulatory relationships of differential coexpression were analyzed. Finally, a total of 181 significant relationship pairs were obtained. The pathways jointly regulated by crosstalk genes and neuropeptides in a significant pair were obtained, and the crosstalk gene-pathway-neuropeptide network was constructed using Cytoscape software (Figure 6(c)). The crosstalk gene-pathway-neuropeptide network showed that the crosstalk genes between MDD and PD could act with neuropeptides indirectly.

### 3.6. Screening and Analysis of Neuropeptides

**Figure 7:**
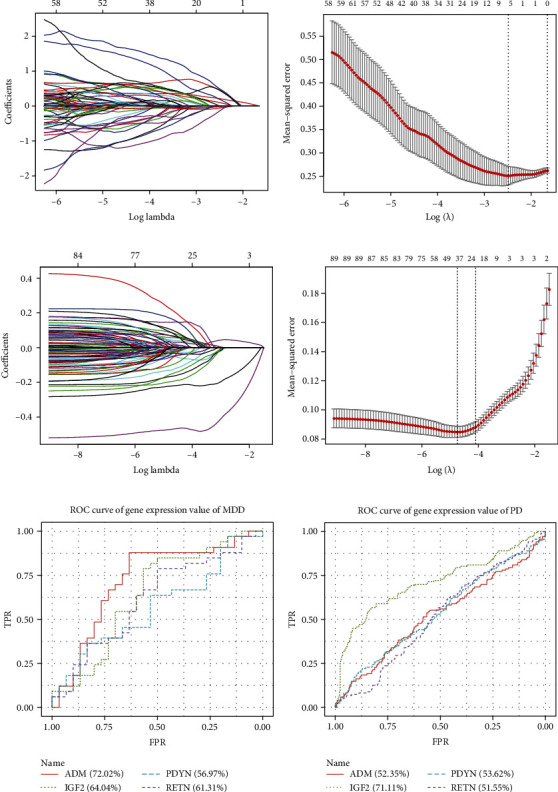
Lasso regression, modeling results, and CV search for the best lambda value. (a) Modeling results of neuropeptides in MDD Lasso regression analysis. The abscissa is log (lambda), and the ordinate corresponds to the correlation coefficient of the modeling process. (b) Neuropeptide screening the relationship between lambda value and mean square error in MDD Lasso regression analysis. The abscissa is log (lambda) and the ordinate is mean square error. There are two dashed lines in the figure, one is the value of *λ* with the minimum mean square error and the other is the value of *λ* with the standard error from the minimum mean square error. (c) Modeling results of neuropeptides in PD Lasso regression analysis; (d) neuropeptide screening lambda value and mean square error in PD Lasso regression analysis. (e) ROC results of ADM, IGF2, PDYN, and RETN in MDD; (f) ROC results of ADM, IGF2, PDYN, and RETN in PD.

The expression profiles of 102 neuropeptides in MDD and PD were obtained and the specific neuropeptides were screened by lasso regression (Figure 7).

Through lasso regression, 9 neuropeptides in MDD and 43 neuropeptides in PD were obtained. Four intersected genes were obtained, i.e., ADM, IGF2, PDYN, and RETN. The expression values of these four genes in MDD and PD were extracted, and ROC analysis of single genes was performed (Figures 7(e) and 7(f)) to check the accuracy of the expression values of these genes at the expression level. The results showed that IGF2 was highly predictive in MDD and PD. ADM was better than the other three genes in predicting MDD disease.

**Figure 8:**
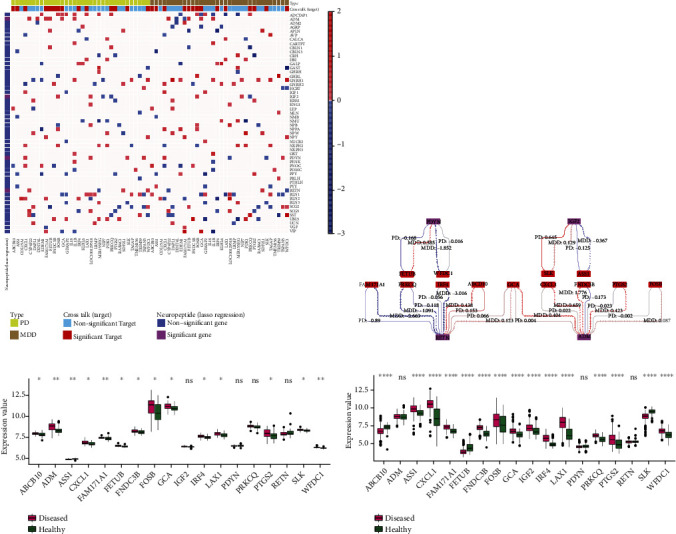
Analysis of crosstalk genes differentially coexpressed with specific neuropeptides. (a) Heat map of highly correlated differential coexpression regulation of crosstalk genes and neuropeptides. (b) Pairs of differential coexpression strength of crosstalk genes differentially coexpressed with specific neuropeptides. The thicker the line, the greater the intensity of regulation; the value on the line represents the specific intensity of regulation; the positive value indicates that the relationship has a consistent trend of regulation in diseases and normal tissues; the negative value indicates inconsistent trend of regulation. (c) Expression of crosstalk genes, which were differentially coexpressed with specific neuropeptides in MDD. In the figure, the relationship between *p* and ^∗^ is as follows: ns indicates *p* > 0.05, ∗ represents *p* ≤ 0.05, ∗∗ represents *p* ≤ 0.01, ∗∗∗ represents *p* ≤ 0.001, ∗∗∗∗ means *p* ≤ 0.0001. (d) Expression of crosstalk genes, which were differentially coexpressed with specific neuropeptides in PD.

According to the differential coexpression regulatory relationship, the DRL of highly correlated coexpression relationships between crosstalk genes and neuropeptides was obtained (Figure 8(a)). Then, the crosstalk genes were analyzed together with these four neuropeptide genes (Figure 8(b)). A total of 13 crosstalk genes were differentially coexpressed with four neuropeptides. The expression profiles of these 13 genes in MDD and PD were extracted, and the expressions of these crosstalk genes in MDD and PD were analyzed (Figures 8(c) and 8(d)), showing that FOSB was highly expressed in MDD and PD.

**Figure 9:**
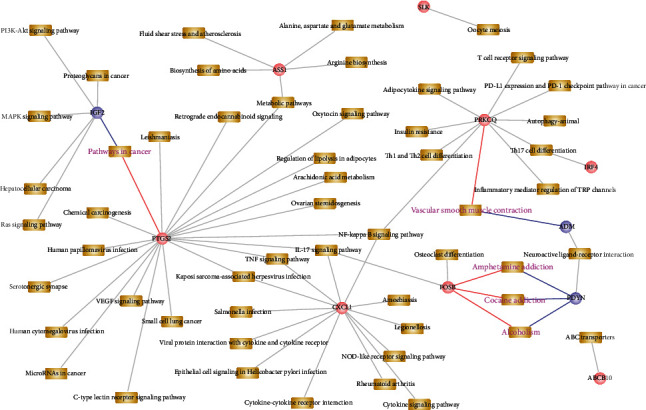
Pathways involved in crosstalk genes differentially coexpressed with ADM, IGF2, PDYN, and RETN. The blue dots represent the four neuropeptides, the pink dots represent crosstalk genes, and the yellow box represents the pathways that genes are associated with.

In addition, pathways related to ADM, IGF2, PDYN, and RETN were extracted and pathways shared by neuropeptides and crosstalk genes were analyzed based on pathway genes (Figure 9).

Figure 8(b) shows that ADM and RETN are differentially coexpressed with multiple crosstalk genes. In the functional analysis, ADM was mainly involved in neuroactive ligand-receptor interaction and vascular smooth muscle contraction. ADM indirectly interacts with FOSB through the neuroactive ligand-receptor interaction pathway, thus affecting the abnormalities of biological processes. ADM and FOSB were differentially coexpressed at the gene expression level and had opposite regulation patterns on MDD and PD in general. In addition, FOSB also regulates the IL-17 signaling pathway. IGF2 regulated the MAPK signaling pathway, PI3K-Akt signaling pathway, and Ras signaling pathway. In the differential coexpression analysis, it was found that IGF2 and ASS1 were highly coexpressed and the regulatory trends were inconsistent between MDD-diseased and healthy control groups as well as PD-diseased and healthy control groups. From the function diagram, it can be seen that ADM, IGF2, PDYN, and RETN can indirectly affect the potential biological function between MDD and PD disease through the function of multiple crosstalk genes.

## 4. Discussion

The neuropeptide genes ADM, IGF2, PDYN, and RETN were found to be intersected between PD and MDD. FOSB was significantly coexpressed with these neuropeptides. Relevant pathways for these genes were the IL-17 signaling pathway, MAPK signaling pathway, Pi3K-Akt signaling pathway, Ras signaling pathway, Neuroactive Ligand-receptor interaction, and vascular smooth muscle contraction.

This is the first bioinformatics study, investigating the crosstalk genes and related neuropeptides between PD and MDD. A relationship between PD and MDD has been extensively discussed in literature, whereby a relationship between these two diseases appear probable [6, 9–12]. The causal link between those two diseases was supposed to be within PD-induced systemic inflammation, leading to a neuroinflammation due to the expression of proinflammatory cytokines [13, 14]. This current study revealed several neuropeptide genes on the transcriptomic level, which will be discussed in the following.

Adrenomedullin (ADM) is a peptide hormone with important roles in the regulation of the cardiovascular and lymphatic systems [17]. ADM was revealed to be a potential biomarker and candidate for therapeutic interventions [17]. A study investigating tissue punches from dentate gyrus revealed ADM as an inhibitor of angiogenesis to be related to neuroinflammation in patients with MDD [18]. Another study revealed that increased levels of ADM and NO in serum of patients would be associated to MDD and related psychomotor retardation [19]. The ADM-NO axis was also elevated due to periodontal pathogenic bacteria, especially *Aggregatibacter actinomycetemcomitans* [20]. Similarly, another study found the ADM-NO-axis to be a functional linkage to PD severity [21]. A lack of sensitivity to ADM could also be related to the bacterial invasion of *Porphyromonas gingivalis*, another periodontal pathogenic bacterium [22]. Furthermore, ADM was found to affect the therapeutic efficiency of the antidepressant paroxetine [23]. Altogether, ADM and the ADM-NO-axis support the role of neuroinflammation as well as the potential relevance of oral pathogens in the relationship between PD and MDD.

Insulin-like growth factor 2 (IGF2) is a hormone regulating cell proliferation, migration, differentiation, and survival [24]. IGF2 was found to mediate depressive behaviors in the brain of rats [25]. Variable methylation of IGF2 was found to be related to the clinical manifestation of MDD in monozygotic twins [26]. Additionally, IGF2 binds on insulin receptor in the brain, whereby dysregulation of IGF2 leads to neuropathological processes [27]. Alongside with IGF1, IGF2 was found to be associated to neuroinflammatory processes [28]. In this respect, insulin-like growth factor 2 mRNA-binding protein 1 was found to promote the NF-*κ*B signaling way, what is induced by lipopolysaccharides, which are important virulence factors of periodontal pathogenic bacteria [29]. This supports the hypothesis of the role of PD in inducing neuroinflammation related to MDD.

Prodynorphin (PDYN) is an endogenous agonist of the k-opioid receptors, having modulatory effects related to addiction; the expression of this peptide is altered in the brain of patients with mental disorders [30, 31]. It has been shown that an impairment of PDYN in the amygdala is associated with MDD [32]. Until now, there is no study reporting the potential relevance of PYDN in PD, making a discussion of this issue difficult.

Resistin (RETN) is a peptide that is secreted by adipocytes, playing roles in metabolism [33]. As an adipokine, RETN is well known to play an important role in development of MDD, while a recent meta-analysis showed that the serum level of resistin was lower in individuals with MDD compared to healthy participants [34]. In patients with MDD, RETN was found to be associated to free cortisol concentrations and therapy outcome [35]. Thereby, a link between obesity and MDD was reported [36]. Moreover, RETN is involved in the interrelationship between MDD and diabetes, whereby neuroinflammation was reported to be of certain relevance [37]. This is a potential and interesting connecting point of MDD and PD; RETN was found to be elevated in gingival crevicular fluid and serum of patients with PD, although this was not related to systemic inflammatory diseases [38]. Another review article showed RETN to be a potential biomarker in the interrelationship between PD and diabetes [39]. In this axis, a causal relation between MDD and PD can be supported.

Lastly, FOSB was significantly coexpressed with these neuropeptides. FOSB is a member of the Fos family of transcription factors and thereby a regulator of stress and antidepressant response [40]. The induction of FOSB in the hippocampus was reported to be critical in addiction and MDD [41]. A network analysis showed that FOSB was one hub gene for depressed suicide [42]. For PD, an analysis based one gene expression data found FOSB to be an inflammation-related gene that might be involved in the development and progression of PD [43]. Accordingly, an influence of this gene on the relation between PD and MDD appears conceivable. Altogether, the hypothesis of neuroinflammation as well as the effect of cofactors like diabetes and obesity in the interplay between PD and MDD can be supported by the bioinformatics data within the current study. Accordingly, interdisciplinary therapeutic and preventive approaches would be needed to comprehensively manage the complex problem of patients suffering from those diseases.

This is the first bioinformatics study investigating the crosstalk and related neuropeptides between PD and MDD. The methodology was comprehensive and revealed a variety of results. However, the bioinformatics approach has several limitations, especially the missing validation of the findings. This must be recognized in the interpretation of the findings. Moreover, there are no data available regarding the included patients. Different patients with MDD and PD were included in this analysis, and thereby, the cohort could be very heterogeneous. Based on these limitations, clinical studies, which evaluate the findings on patients suffering from MDD and PD, are needed. As long as these findings are not available, the results and respective conclusions of this bioinformatics study remain speculative. Thereby, it must be recognized that all findings in the current study were only revealed on the transcriptomic level.

## 5. Conclusion

The neuropeptide genes ADM, IGF2, PDYN, and RETN were found to be intersected between PD and MDD, and FOSB was a crosstalk gene, which was related to these neuropeptides on the transcriptomic level. These findings could be a basis for future research in the field, needing further validation.

## Figures and Tables

**Table 1:** Datasets for analysis.

Disease	Series	Platforms	Case	Control	Total
**MDD**	GSE38206	GPL13607	9	9	18
	GSE39653	GPL10558	21	24	45

**PD**	GSE10334	GPL570	183	64	247
	GSE16134	GPL570	241	69	310
	GSE23586	GPL570	3	3	6

**Table 2:** Statistics of differentially expressed genes.

Disease	Up	Down	Total
MDD	674	237	911
PD	664	461	1125

**Table 3:** Topological properties of top 20 genes.

Name	Label	Degree	Average shortest path length	Betweenness centrality	Closeness centrality	Topological coefficient
UBC		418	2.339286	0.553074	0.427481	0.056261
APP		64	3.125	0.160397	0.32	0.114833
ELAVL1		64	2.946429	0.157445	0.339394	0.119835
NUCB2	Neuropeptide	50	3.169643	0.032524	0.315493	0.233333
DBI	Neuropeptide	48	3.241071	0.017919	0.30854	0.291667
UBL5	Neuropeptide	42	3.098214	0.018099	0.322767	0.344828
TAC3	Neuropeptide	42	3.276786	0.020327	0.305177	0.356322
CHGA	Neuropeptide	42	3.276786	0.013341	0.305177	0.516667
SCT	Neuropeptide	38	3.330357	0	0.300268	0
SCG2	Neuropeptide	38	3.330357	0	0.300268	0
PENK	Neuropeptide	38	3.330357	0	0.300268	0
CCK	Neuropeptide	38	3.330357	0	0.300268	0
ADIPOQ	Neuropeptide	38	3.330357	0	0.300268	0
GAL	Neuropeptide	22	3.723214	0.011257	0.268585	0.388889
PTGS2	Crosstalk	18	2.758929	0.16726	0.36246	0.133333
PTHLH	Neuropeptide	18	3.848214	0.005528	0.259861	0.5
CHGB	Neuropeptide	18	3.401786	0.060159	0.293963	0.166667
PDK1	Crosstalk	16	2.830357	0.115809	0.353312	0.146429
IL1B	Crosstalk	16	3.0625	0.190297	0.326531	0.132813
MYC		16	3.160714	0.0628	0.316384	0.231579

## Data Availability

The datasets used and/or analyzed during the current study are available from the corresponding author upon reasonable request.
